# Improving Access to Child and Youth Addiction and Mental Health Services in New Brunswick: Implementing One-at-a-Time Therapy Within an Integrated Service Delivery Model

**DOI:** 10.1007/s11469-024-01339-4

**Published:** 2024-06-24

**Authors:** Laura M. Harris-Lane, Alesha C. King, Stéphane Bérubé, Katie Burke, AnnMarie Churchill, Peter Cornish, Alexia Jaouich, Mylène Michaud, Anne Losier, Jai Shah, Joshua A. Rash

**Affiliations:** 1 Department of Psychology, Memorial University of Newfoundland, 230 Elizabeth Ave, St. John’s, NL A1B 3X9 Canada; 2 Addiction & Mental Health Services, Department of Health, Government of New Brunswick, Fredericton, NB Canada; 3 Stepped Care Solutions, Mount Pearl, NL Canada; 4 Department of Psychiatry, McGill University, Montreal, QC Canada; 5 Quality Improvement, Department of Health, Government of New Brunswick, Fredericton, NB Canada; 6 Organizational Change Management Practice, Mariner Innovations, Halifax, NS Canada

**Keywords:** Child, Adolescent, Mental health services, Implementation science, Single session therapy

## Abstract

This study examines the process of implementing One-at-a-Time (OAAT; i.e., single-session) therapy into child and youth addiction and mental health services in New Brunswick. The implementation process was retrospectively mapped onto implementation science frameworks. Providers were recruited to participate in research associated with the system change, and completed surveys to assess readiness for implementation. Client satisfaction and system-outcomes were measured through surveys and system indicators, respectively. Key implementation considerations included age of consent for services, implementation within an integrated service delivery model, and mapping the client journey to conceptualize changes in service delivery. Providers (*N* = 214) felt that OAAT therapy was acceptable and appropriate to implement into practice, and would lead to observable short-term outcomes. During the implementation (April–December 2022), 2266 sessions were delivered, resulting in a 62% waitlist reduction. Most clients who completed the satisfaction survey (*N* = 518) reported benefit. This study elucidates the successful implementation of OAAT therapy for children and youth, and can serve as a heuristic for comparable practice change initiatives.

**Supplementary Information:**

The online version contains supplementary material available at 10.1007/s11469-024-01339-4.

Evidence suggests that 20% of Canadian children and youth live with a mental health disorder (Georgiades et al., 2019), with global estimates that only 43% of young people in high-income countries receive treatment (Friedman et al., 2015). Low mental health literacy, stigma, and limited availability of services have been cited as the most prevalent barriers to accessing services (Moroz et al., 2020; Radez et al., 2021). The average wait time to access child and youth addiction and mental health services (A&MHS) in Canada’s largest province was 67 days in 2020 (Children's Mental Health Ontario, 2020), with one Canadian province reporting a 33% increase in demand for services among adolescents between 2015 and 2020 (Government of New Brunswick, 2021). Challenges in accessing timely services have resulted in increased demands on Emergency Departments, as visits for child and youth mental health and addiction concerns increased by almost 90% between 2007 and 2017 (Chiu et al., 2020). Recent qualitative research on youth and parent experiences accessing mental health and addiction services in Canada highlighted the frustrations and toll of fragmented services with protracted wait times (Kourgiantakis et al., 2023; Zifkin et al., 2021).

The long-term impacts of unaddressed mental health concerns in childhood can have detrimental impacts on education attainment (Breslau et al., 2008), engagement in higher-risk behaviors (e.g., sexual health; Harmanci et al., 2023), disability-adjusted life years (Patel et al., 2007), criminal justice-involvement (Beaudry et al., 2021), and risk of suicide (Bilsen, 2018). In 2023, Canadian economists estimated that the investment in child and youth mental health would result in savings of $30 billion per annum (The Conference Board of Canada, 2023). It is imperative that A&MHS are accessible and effective given the significant and increasing burden associated with mental health disorders among children and youth (Piao et al., 2022).

The New Brunswick Department of Health reviewed community A&MHS to create a 5-year interdepartmental action plan that identified five key areas: (1) increasing access to care; (2) appropriately matching individuals to care; (3) improving population health; (4) providing earlier intervention; and (5) reducing substance-related impacts (refer to Supplemental Appendix A; Government of New Brunswick, 2021). Improving timely access to A&MHS was identified as an immediate provincial priority. After reviewing various frameworks, the Government of New Brunswick decided to co-design and adopt a provincial stepped care model for the delivery of A&MHS using Stepped Care 2.0 (SC2.0; Cornish, 2020). Moreover, implementing a One-at-a-Time (OAAT) approach (akin to single-session therapy and a core component of the SC2.0 model) in community A&MHS was identified as the best course of action to improve access to care and reduce wait times.

SC2.0 is a flexible and collaborative model of A&MHS delivery that is guided by a series of key principles, including prioritizing informed choice, and the preferences, readiness, and needs of clients (Fig. 1). As a planning tool for system change, the SC2.0 model organizes resources and supports along hierarchical steps that range from low- to high-intensity, requiring varying levels of investment by service users and stakeholders (Fig. 2). The SC2.0 model is presented to clients as a continuum of care, including formal and informal resources, to best meet their preferences, readiness, and needs. Clients and providers can collaboratively adjust the intensity level and types of care received through informed decision-making (Cornish, 2020). Additionally, clients can simultaneously avail of supports and resources at varying intensities (e.g., self-help and peer support) (Cornish, 2020). A systematic review and meta-analysis on the usage of mental health care options based on client preferences suggested that clients have increased engagement and therapeutic alliance when using their preferred mental health and addiction treatment (Windle et al., 2020), which can have significant impacts on client outcomes (Baier et al., 2020).

**Fig. 1:**
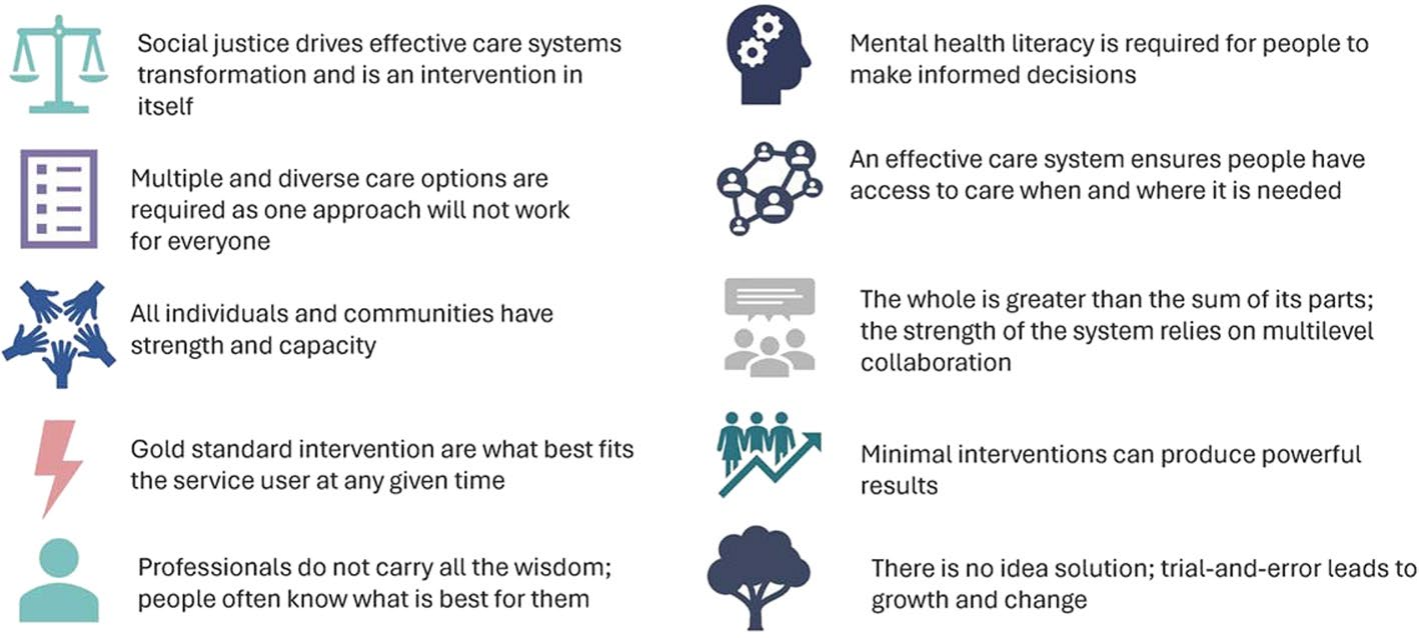
Guiding principles of the Stepped Care 2.0 model

**Fig. 2:**
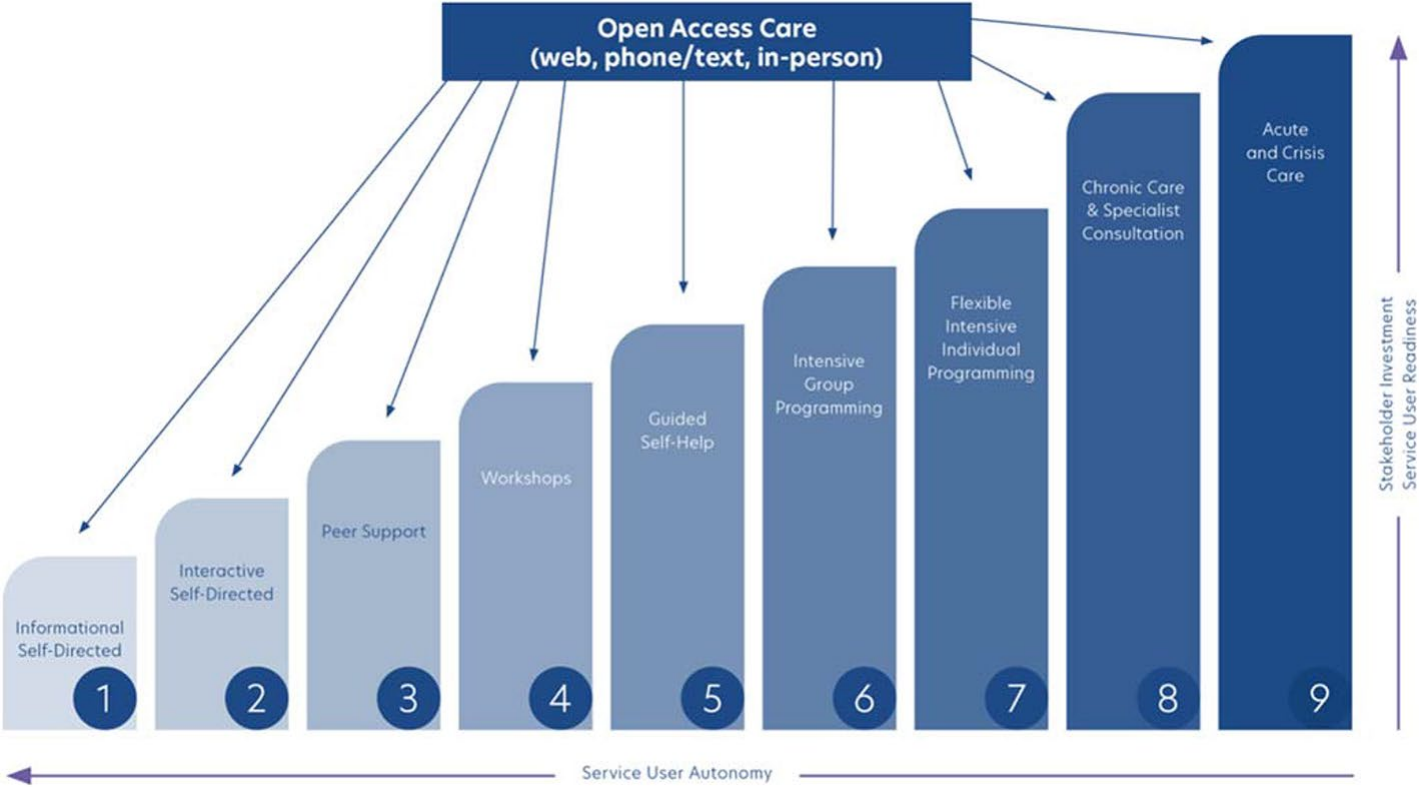
Stepped Care 2.0 planning tool for system change

This model was chosen due to strong face validity, allowance for a comprehensive and recovery-oriented continuum of services with varying levels of intensity, focus on rapid access to care, and promotion of evidence-based practices (Carey et al., 2021). The SC2.0 model contains nine core components depicted in Fig. 3. The first five components of the SC2.0 model focus on system design and improvement, while the remaining four core components center around the client’s care.

**Fig. 3:**
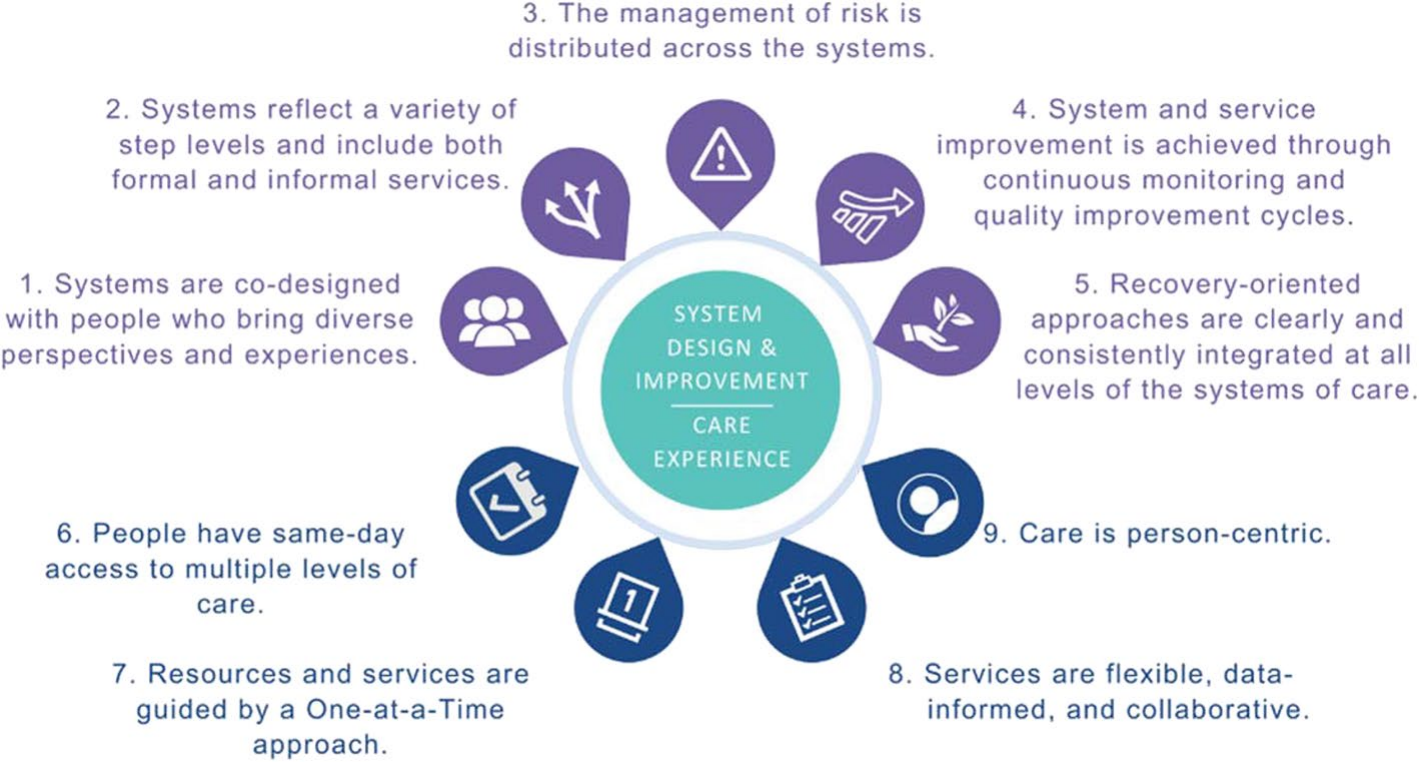
Nine core components of the Stepped Care 2.0 model

An OAAT therapeutic approach, often referred to as single-session therapy, focuses on the client’s top-of-mind concern. Each client encounter is treated as a whole, leverages a solution-focused and strengths-based approach, and provides an opportunity for hope, growth, and change (Hair et al., 2013). A meta-analysis of 50 randomized controlled trials reported that OAAT therapy (i.e., single-session therapy) resulted in significant improvements in psychological concerns of children and youth, with a moderate effect size (g = 0.32; Schleider & Weisz, 2017). Specifically, an OAAT therapeutic approach was beneficial for a variety of presenting concerns (e.g., anxiety, conduct disorder, self-esteem) at varying levels of severity, and in community samples. Children and youth that received an OAAT intervention had an increased likelihood of reporting improved outcomes compared to a control group (Schleider & Weisz, 2017). These findings, paired with other research on using OAAT approaches as a mechanism to increase timely access to care (i.e., reducing waitlists and wait times; Harris-Lane et al., 2023; Thomas et al., 2021), continue to support utility of implementing OAAT therapy in New Brunswick's child and youth community A&MHS. As a first step to implementing a provincial SC2.0 model, OAAT therapy was implemented within child and youth A&MHS, which are delivered through an integrated service delivery model (Settipani et al., 2019). Integrated service delivery models, such as Integrated Youth Services, have gained traction internationally (i.e., Australia, Ireland, England), with the aim of integrating often-siloed sectors (McGorry et al., 2013). The New Brunswick integrated service delivery model (Government of New Brunswick, 2015) includes a partnership between Health, Education and Early Childhood Development, Social Development, and Justice and Public Safety. Services are offered in school- and community-based settings, with the goal of eliminating gaps in service delivery, fostering a growing environment suitable for children and youth with various needs, and developing interventions tailored to the needs and preferences of children and youth. Services included in the New Brunswick integrated service delivery model include OAAT therapy, individual and group counseling, substance use and gambling support, mental wellness education, navigation for school supports and resources, and partnerships to facilitate referrals to out-of-home care (e.g., inpatient treatment, child protection placement) where appropriate. The collaborations between the four sectors enhance the delivery of services for children and youth, as the interdisciplinary team meets regularly (e.g., weekly) to discuss cases. Children, youth, and families are involved in all aspects of the planning, decision-making, and follow-up (Government of New Brunswick, 2015).

## Rationale and Objectives

This manuscript illustrates the steps taken to implement OAAT therapy within New Brunswick child and youth A&MHS. We aim to highlight the following: (1) key implementation considerations; (2) readiness among providers to implement OAAT therapy within a SC2.0 continuum; and (3) client satisfaction, and system-related outcomes associated with implementing OAAT therapy.

## Methods

Ethical approval was sought and received from the Horizon Health Network ethics board (Ref# 2021–3015), Vitalité Health Network ethics board (Ref# 2957), and Newfoundland and Labrador Health Research Ethics Board (Ref# 2021.094). Participants provided informed and written consent before participating in the associated research. Participation was voluntary, data were collected by research assistants who were not affiliated with participating healthcare networks or school districts, and only aggregate data were shared with the government.

### Procedures

#### Key Implementation Considerations

The process of implementing OAAT therapy into child and youth A&MHS was documented through meeting minutes recorded from weekly and bi-weekly meetings, project charters (i.e., a living document updated regularly), staffing notes, themes arising from check-ins with program managers and clinical leads, and government presentations. Changes to implementation plans, as well as successes and challenges, were identified through documented consultations with the core project team. Processes, contextual factors, implementation determinants, and implementation strategies were identified, and retrospectively mapped onto the Active Implementation Framework–Implementation Stages (AIF-IS; Fixsen et al., 2005), Consolidated Framework for Implementing Change (CFIR; Damschroder et al., 2022), and Expert Recommendations for Implementing Change (ERIC; Powell et al., 2015).

#### Readiness Among Providers to Implement OAAT Therapy

Providers who worked in child and youth A&MHS in Horizon Health Network, Vitalité Health Network, and the seven School Districts were recruited to participate between October 2021 and April 2022. Study procedures mirrored those detailed in our study on implementing OAAT therapy in adult A&MHS (Harris-Lane et al., 2023). Child and youth A&MHS providers received communications from management (i.e., via email, staff meetings) that outlined the initiative to implement OAAT therapy within the context of a provincial SC2.0 model. Providers were directed to a consent form on Qualtrics to learn more about the optional research being conducted as part of this initiative. All child and youth A&MHS providers, from varying professional backgrounds, were eligible to participate. Those who consented to participate completed a demographic survey (T1) before receiving access to an online SC2.0 training course, followed by an OAAT therapy course. Providers who elected not to participate were immediately redirected to the training courses. The SC2.0 and OAAT therapy courses (detailed in Supplemental Appendices B and C, respectively) spanned 3–5 h, and contained videos, case studies, and self-reflection journal entries. After completing the asynchronous courses, participants completed a post-course survey (T2) on the acceptability, feasibility, and appropriateness of OAAT therapy. Participants completed a 1-month follow-up survey (T3) with a comprehensive measure of organizational readiness. Providers received three $20 e-gift cards for their participation, and had the option to participate in their preferred language (English or French).

#### System-Related Outcomes and Client Satisfaction

Providers documented each OAAT therapy session using the provincial Client Service Delivery System (CSDS). Key performance indicators were abstracted from CSDS and standardized manual reports, and included the following: (1) number of OAAT therapy sessions delivered between April and December 2022; (2) provincial waitlists for child and youth A&MHS during the implementation, and 6 months preceding it (i.e., November 2021 to December 2022); and (3) number of clients who returned for multiple sessions during the project implementation period. Refer to Harris-Lane et al. (2023) for a detailed description of procedures.

Child and youth clients who availed of an OAAT therapy session were offered the opportunity to complete a satisfaction survey by administrative or clinical staff. Parent or guardian consent for participation was obtained for clients under the age of 16. Surveys were completed in approximately 5 min and were administered between April and December 2022.

### Measures

#### Key Implementation Considerations

Data were collected from a variety of sources within the child and youth A&MHS, including (1) government reports and presentations, (2) field notes and meeting minutes, (3) documented discussions on implementation progress and areas of improvement, (4) staff charting and notes, and (5) documented consultation with core child and youth project team.

#### Readiness Among Providers to Implement OAAT Therapy

Acceptability, appropriateness, and feasibility were assessed using the Acceptability of Intervention Measure (AIM), Intervention Appropriateness Measure (IAM), and Feasibility of Intervention Measure (FIM), respectively (Weiner et al., 2017). The AIM, IAM, and FIM each contain four items with scores ranging from 1 (strongly disagree) to 5 (strongly agree). Higher mean scores suggested greater acceptability, appropriateness, and feasibility.

Organizational readiness for implementation was assessed using the Readiness Diagnostic Scale (RDS), adapted by the developers of the Readiness for Integrated Care Questionnaire for use in this project (Scott et al., 2017). The RDS contains 51 items and 18 subscales (refer to Supplemental Appendix D). Scale anchors range from 1 (strongly disagree) to 7 (strongly agree), with higher mean scores suggesting greater readiness for implementation.

#### System-Related Outcomes and Client Satisfaction

Data on the number of OAAT therapy sessions delivered, discrete client visits, and waitlists were abstracted from the CSDS and standardized manual reports. The client satisfaction survey used in the implementation of OAAT therapy for adults (Harris-Lane et al., 2023) was adapted for use with children and youth (refer to Supplemental Appendix E). The adapted survey included 5 items on a 5-point Likert scale, whereby satisfaction was depicted using images of facial expressions ranging from “very upset” to “not upset.” These “smiley meters” have considerable validity and reliability in data collection with child and youth populations (Read et al., 2002). The client satisfaction survey also included an open-ended question, where clients could provide written feedback on their experience. Demographic information was not collected for confidentiality purposes.

### Data Analysis

#### Key Implementation Considerations

Initiatives that occurred throughout the implementation process were mapped onto corresponding AIF-IS elements, CFIR domains, and ERIC strategies using a four-step process. First, the research team reviewed documentation and created a timeline of New Brunswicks’s actions and processes of implementing OAAT therapy into child and youth A&MHS. Second, the timeline was reviewed and refined by members of the core project team for accuracy. Third, two members of the research team (LH-L and JR) reviewed component definitions within the AIF-IS, CFIR, and ERIC strategies to determine how steps undertaken within the OAAT implementation steps aligned with each framework. Finally, the implementation process with retrospectively mapped components was reviewed by the broader team for consensus.

#### Readiness Among Providers to Implement OAAT Therapy

Descriptive statistics (i.e., frequencies, arithmetic means, standard deviations) were conducted using IBM SPSS V28 to characterize the sample and understand providers’ perceptions of readiness for implementing OAAT therapy. Data were missing at random, and were not imputed due to the descriptive nature of analyses.

#### System-Related Outcomes and Client Satisfaction

Client satisfaction with OAAT therapy sessions and system performance indicators were analyzed using descriptive statistics. Client responses to the written survey question were coded and synthesized thematically (Braun & Clarke, 2006). Emerging themes were developed from patterns across participant responses in an iterative process and the coding scheme used was equivalent to coding performed for surveys administered to clients accessing adult services (Harris-Lane et al., 2023).

### Role of Funding Source

This work was supported by the Canadian Institutes of Health Research (CIHR) under the Transitions in Care Team Grant No. 423968. The funding source was not involved in the project design, conduct, or reporting.

## Results

### Key Implementation Considerations

The implementation of OAAT therapy into child and youth A&MHS followed a similar approach to the implementation process documented within adult A&MHS (Harris-Lane et al., 2023). The unique complexities of implementing services for children and youth, and particularly in the context of an integrated service delivery model, necessitated unique considerations and adaptations. The full implementation process as aligned to the four stages of the AIF-IS, CFIR determinants, and ERIC strategies is detailed in Supplemental Appendix F. For brevity, we only describe the key learnings and implementation considerations unique to child and youth A&MHS within the installation and initial implementation stages.

#### Installation

The installation stage offered a unique opportunity to leverage the successes and learnings from the implementation of OAAT therapy in adult A&MHS, and adapt previous efforts to fit the child and youth A&MHS context. The installation stage commenced by hiring a senior health consultant in October 2021 who oversaw the implementation and helped sustain the delivery of OAAT therapy. The senior health consultant was responsible for conducting assessments of risk and readiness throughout the project and liaised with implementation leads.

Between 2021 and 2022, the province followed a staffing plan and hired eight OAAT clinical leads (i.e., implementation leads) who acted as champions in supporting and sustaining the implementation. These clinical leads provided clinical supervision to providers, and served as a vital connection between the 44 teams and the senior health consultant. Each clinical lead also delivered OAAT therapy sessions to enhance expertise and gain personal experience with the successes and challenges of this form of service delivery. The core project team cited that the unique perspectives of clinical OAAT leads—based on their own delivery of service and consultations with providers—were critical in the implementation and sustainability of OAAT therapy. Further, additional 18.5 full-time equivalent positions were created for the delivery OAAT therapy.

**Fig. 4:**
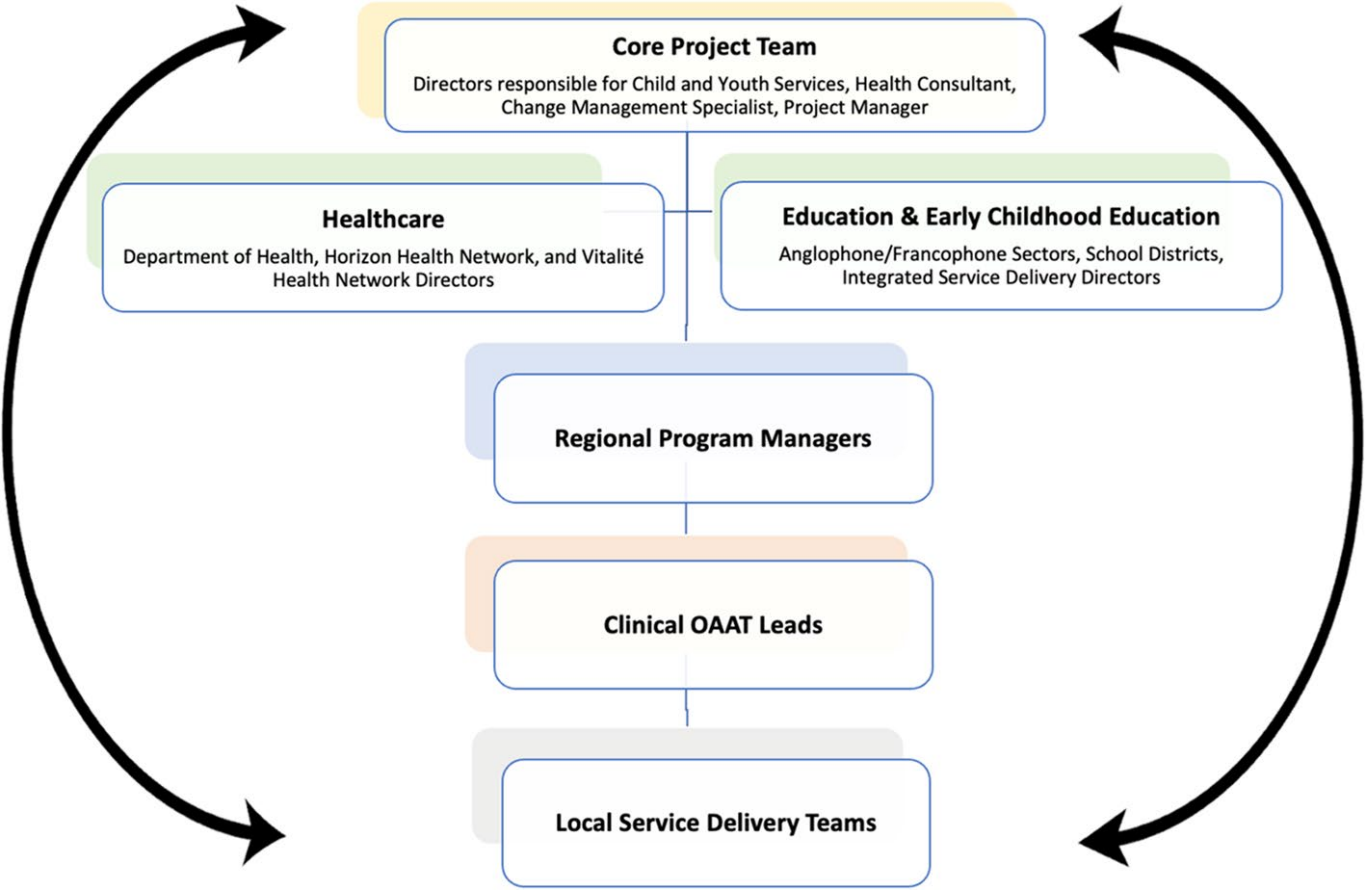
Flow of information from core project team to providers implementing OAAT therapy in their practice. *Notes*: The Provincial Working Group included (1) the core project team, (2) directors in both healthcare and education and early childhood education sectors, (3) regional program managers, and (4) OAAT clinical leads

The provincial working group (i.e., implementation team) was established in October 2021 and included membership from the Department of Health, Directors from Horizon and Vitalité Health Networks, child and youth A&MHS Managers, and Education and Early Childhood Development Directors. Clinical OAAT leads were onboarded to the working group as they were hired. Similar to the implementation of OAAT therapy into adult A&MHS (Harris-Lane et al., 2023), feedback loops were established between stakeholders who had a vested interest in the success of the implementation; refer to Fig. 4. The provincial working group met weekly to (1) adapt the implementation plan created for implementing OAAT therapy into adult A&MHS, (2) revise operational guidelines to best fit processes of delivering OAAT therapy in both adult and child and youth A&MHS, and (3) review successes, challenges, and outcomes of ongoing readiness assessments.

Managers and clinical leads completed asynchronous online courses in SC2.0 and OAAT therapy beginning in November 2021, with providers following shortly thereafter. Approximately 400 healthcare and education providers were trained in this form of service delivery, with 214 (54%) enrolling in the associated research. Additionally, providers completed a live OAAT therapy training with an expert in OAAT approaches in child and youth A&MHS. While some providers expressed hesitancy towards the change in service delivery, completion of online and live courses and support from clinical leads were critical in helping alleviate concerns.

#### Initial Implementation

Child and youth teams trialed integrating OAAT therapy into practice in November 2021, with an expectation that all teams would offer OAAT therapy by May 2022. Readiness assessments were conducted with the manager of each team between October and November 2021 to mark the beginning of implementing OAAT therapy into practice. These assessments were used to explore operational approaches, challenges, and priorities. Assessment results highlighted four key challenges: (1) staff capacity (*n* = 35); (2) availability of physical space (*n* = 27); (3) age of consent (*n* = 19); and (4) integration with integrated service delivery processes (*n* = 13).

Concerns surrounding staff capacity were not unique to the implementation of OAAT therapy in child and youth A&MHS (Harris-Lane et al., 2023); however, limited access to available physical space presented a particular challenge. Adult A&MHS operated in designated clinics while child and youth teams delivered services within schools, often without reserved space. Notably, the ability to allocate space for OAAT therapy sessions in child and youth A&MHS was beyond the decision-making power of leadership on the provincial working group. In response, the core project team formed a task force with senior members of government leadership in healthcare and education.

Existing integrated service delivery processes created additional complexity and required adaptation for the successful integration of OAAT therapy into practice. For instance, prior to implementing OAAT therapy, child and youth teams would meet with integrated service delivery partners to review new referrals before contacting the client. In these meetings, the team would discuss if an intake assessment for services would be offered. However, following implementation, OAAT therapy sessions were established as a standard first point of contact, and integrated service delivery consultation meetings were held on an as-needed basis after this initial therapeutic intervention. The core project team completed a client journey mapping exercise with managers, clinical leads, and providers to facilitate changes in service delivery. This exercise aligned with tools previously used in the research (Davies et al., 2023; McCarthy et al., 2016), and was used to support providers in conceptualizing the changes, and identify potential barriers and facilitators to offering OAAT therapy sessions within existing policies. Refer to Supplemental Appendix G for further information. During the mapping exercise, providers anticipated that processes for scheduling appointments (e.g., phone calls) may need to be adapted to best meet youths’ preferences (e.g., texting, social media).

In addition to challenges with integrated service delivery processes, healthcare providers, education providers, and community partners experienced early tensions due to the need for an enhanced understanding of the referral process, and of the roles of themselves and their collaborators in the delivery of OAAT therapy. The core project team held education sessions with these stakeholders to clarify roles and expectations, and help mitigate these tensions.

Due to the complexities of child and youth A&MHS delivery, the Government of New Brunswick shifted communications from “open access” or “drop-in services” to highlight the “rapid access,” “low-barrier,” and “early intervention” nature of OAAT therapy. The provincial working group determined that OAAT therapy sessions would be scheduled in advance, with the goal of achieving a maximum wait time of 3 days for scheduled OAAT therapy sessions.

Beginning in April 2022, child and youth teams distributed client satisfaction surveys to inform iterative improvements, and to better understand experiences with the service among children, youth, and parents and guardians.

### Readiness Among Providers to Implement OAAT Therapy

As characterized in Table 1, child and youth providers (*N* = 214) enrolled in this study, with 213 providers completing T2 surveys, and 155 providers completing T3 surveys. There were no significant differences in the demographics of providers from T1 to T3. Among participants, 66.4% indicated English as their preferred language while 33.6% preferred French. Providers worked in the two health authorities, Horizon Health Network (45.3%) and Vitalité Health Network (25.2%), as well as the school districts (29.0%). The majority of providers represented healthcare providers (68.7%) on the integrated child and youth teams, and reported a professional background in social work (61.7%).

**Table 1:** Summary of participant characteristics

	T1	T3	Group differences between T1 and remaining T3 participants	
	Sample size		Chi-square test	
Characteristic	*N* (%)	*N* (%)	χ^2^	*p*
Language			N/A	N/A
English	142 (66.4%)	N/A		
French	72 (33.6%)	N/A		
Organization of work			7.06	.070
Horizon Health Network	97 (45.3%)	75 (48.1%)		
Vitalité Health Network	54 (25.2%)	32 (20.5%)		
School Districts	63 (29.5%)	49 (31.4%)		
Work Setting			1.98	.371
Child and Youth Team (Healthcare)	147 (68.7%)	105 (67.3%)		
Child and Youth Team (Education)	63 (29.4%)	49 (31.4%)		
Other	4 (1.9%)	2 (1.3%)		
Community setting			4.15	.042*
Urban	116 (54.5%)	91 (58.7%)		
Rural	97 (45.5%)	64 (41.3%)		
Profession			8.72	.190
Social work	132 (61.7%)	93 (59.6%)		
Psychology	29 (13.6%)	22 (14.1%)		
Education	13 (6.1%)	13 (8.3%)		
Counselling	12 (5.6%)	9 (5.8%)		
Nursing	12 (5.6%)	7 (4.5%)		
Occupational therapy	9 (4.2%)	8 (5.1%)		
Other	7 (3.3%)	4 (2.6%)		
Level of education			8.80	.117
Doctorate	6 (2.8%)	6 (3.8%)		
Master’s	71 (33.2%)	57 (36.5%)		
Baccalaureate	126 (58.9%)	86 (55.1%)		
Diploma	6 (2.8%)	5 (3.2%)		
Other	5 (2.3%)	2 (1.3%)		
Role			2.26	.520
Provider	166 (77.6%)	123 (78.8%)		
Manager/provider	26 (12.1%)	16 (10.3%)		
OAAT lead	11 (5.1%)	9 (5.8%)		
Manager	11 (5.1%)	8 (5.1%)		

^*^p < .05. ^**^p < .01. ^***^p < .001

*N/A* not applicable, as data were not collected at that timepoint

As detailed in Table 2, providers agreed that OAAT therapy is an acceptable solution to the challenges faced in child and youth A&MHS when delivered within the context of a provincial SC2.0 model (*M*_*AIM*_ = 4.16, *SD*_*AIM*_ = 0.65), and was appropriate for clients, the organization, and their practice (*M*_*IAM*_ = 4.01, *SD*_*IAM*_ = 0.67). Participants somewhat agreed that implementing OAAT therapy was feasible and practical (*M*_*FIM*_ = 3.73, *SD*_*FIM*_ = 0.69).

**Table 2:** Descriptive statistics for measures of acceptability, appropriateness, feasibility, and readiness

Measures and subscales	*M* ± *SD*
Acceptability of Intervention Measure (AIM)	4.16 ± 0.65
Intervention Appropriateness Measure (IAM)	4.01 ± 0.67
Feasibility of Intervention Measure (FIM)	3.73 ± 0.69
Readiness Diagnostic Scale (RDS)	
Compatibility	5.71 ± 0.91
Observability	5.53 ± 0.89
Program champion	5.36 ± 1.42
Priority	5.35 ± 1.14
Culture	5.33 ± 1.04
Simplicity	5.26 ± 1.04
Leadership	5.24 ± 1.18
Climate	5.08 ± 1.10
Supportive climate	5.01 ± 1.30
Innovation specific knowledge and skills	4.99 ± 1.25
Innovativeness	4.96 ± 1.25
Structure	4.95 ± 1.38
Ability to pilot	4.92 ± 1.52
Relative advantage	4.79 ± 1.30
Intra-organizational relationships	4.40 ± 1.49
Staff capacity	4.30 ± 1.37
Resource utilization	4.20 ± 1.58
Inter-organizational relationships	4.16 ± 1.45

Providers noted areas of strength in organizational readiness, including perceptions that OAAT therapy was compatible with their practice, client needs, and the organization’s mandate (*M* = 5.71, *SD* = 0.91). Providers also endorsed the belief that OAAT therapy would lead to observable short-term outcomes (*M* = 5.53, *SD* = 0.89). In contrast, areas of growth included concerns around staff capacity (*M* = 4.30, *SD* = 1.37) and insufficient connections with other organizations that have implemented OAAT therapy (*M* = 4.16, *SD* = 1.45). Table 2 presents a detailed depiction of RDS components measured.

### System-Related Outcomes and Client Satisfaction

**Fig. 5:**
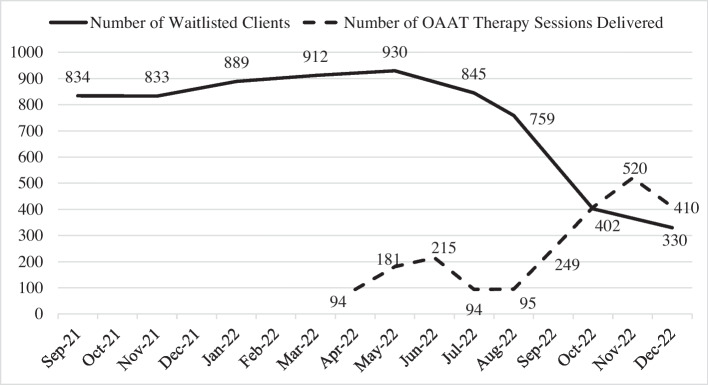
Number of waitlisted clients and OAAT therapy sessions delivered by month. *Note*: implementation period of OAAT therapy occurred between April and December 2022

A total of 2266 OAAT therapy sessions were delivered to clients seeking child and youth A&MHS between April and December 2022. Among the 1676 clients seeking care (April 2022 to December 2022), 88.8% of clients used one session while 9.1% returned for a second session, and 2.1% returned for three or more sessions. Prior to implementation (September 2021–March 2022), on average, 867 clients were waitlisted to receive care. As illustrated in Fig. 5, this number was reduced by 62% to 330 waitlisted clients by December 2022, due to the delivery of OAAT therapy sessions, review and consolidation of waitlists, and transition to CSDS.

Clients (*N* = 518) completed a satisfaction survey following their OAAT therapy session. Before receiving a session, 61% of clients reported feeling upset/very upset about their concerns, with only 7% of clients reporting feeling upset/very upset following the session. Most clients reported feeling confident in their ability to manage their mental health concern after the session (59%), satisfaction in how their concerns were addressed in the session (89%), and satisfaction with the co-developed treatment plan (87%).

As detailed in Table 3, five themes emerging from client (*n* = 104) comments. Specifically, clients felt that (1) the OAAT therapy session was a positive experience characterized by a strong therapeutic alliance with the provider, and resulted in enthusiasm towards their recovery (*n* = 49), (2) the session equipped them with knowledge and tools that can be applied in their day-to-day life (*n* = 17), (3) the OAAT therapy session was valued and appreciated (*n* = 26), (4) they experienced improved wellbeing following the session (*n* = 13), and (5) the OAAT therapy session did not match the clients’ expectations of care, or adequately meet their needs at that time (*n* = 6).

**Table 3:** Results of thematic analysis derived from child and youth client satisfaction survey comments

Description of finding	Supporting quotation	Number of endorsements (*N* = 104)
Theme 1: Clients had a positive experience with their OAAT therapy session		
Clients described their OAAT therapy session as a positive experience. Some clients indicated enthusiasm towards their recovery, and felt they had a strong therapeutic alliance with the provider	*I’m quite excited to see how this’ll go for me. And I’m eternally appreciative of [the clinician's] support and guidance she has given me*	*n* = *49*
	*[Provider] has great demeanor to talk with our teenager, she was great delivering the message and providing guidance*	
	*This was a good experience and while I didn’t exactly g[e]t what I was expecting it is very good and I would like to keep coming here*	
Theme 2: Clients felt equipped with resources to address their concern		
Clients felt that the session facilitated the development of a treatment plan, and equipped them with knowledge and tools that can be applied in their day-to-day life	*This is the first time I have left with a solid plan with timelines and objectives, [the clinician] was able to really identify succinctly where help was needed*	*n* = *17*
	*Liked how he listened to me and gave me really good ideas on how to work on my anxiety. We came up with a good plan*	
Theme 3: Clients perceived value in attending an OAAT therapy session, and were appreciative of what OAAT sessions can offer		
Clients expressed value and appreciation for the OAAT therapy session	*Very glad that this program is available*	*n* = *26*
	*Appreciate the interest in having an appointment and that this meeting included all family members (parents and my son)*	
Theme 4: Clients felt that the OAAT therapy session helped improve their well-being and recovery journey		
Clients reported improved wellbeing immediately after attending the OAAT therapy session. Sessions were described as helpful in developing client’s confidence and resolving concerns	*This was extremely beneficial. I appreciate being able to talk to someone & to have a course of action*	*n* = *13*
	*It was great to sit and talk to someone in-depth about my problems and concerns ve[r]y thankful for a long “vent session” would recommend to anyone nee[d]ing advice or someone to just listen and talk to if needed in the moment*	
	*This meeting gave our daughter confidence that she is taking steps to improve the obstacles in her daily life*	
Theme 5: Client’s needs or expectations were not met during the OAAT therapy session		
A minority of clients reported that the OAAT therapy session did not match their expectations of care, and did not believe the session had adequately addressed their needs at that moment	*Felt like the advice my friends would give me and didn’t actually help*	*n* = *6*
	*Not enough resources. No set plan continuous help/counselling*	

## Discussion

Following the rollout of the integrated service delivery model in 2015 and 2016, the province of New Brunswick experienced a 33% increase in referrals for child and youth A&MHS (Government of New Brunswick, 2021). The Government of New Brunswick made it a priority to increase rapid access to care through the adoption of OAAT therapy, and work toward a provincial SC2.0 model. The implementation of OAAT therapy into child and youth A&MHS presented challenges due to the lack of literature on pragmatic processes and considerations for implementing OAAT therapeutic approaches with this population, particularly considering unique needs, such as parent/guardian consent. As such, we aimed to begin to address this gap by documenting the process of implementing OAAT therapy within child and youth A&MHS, detailing initial results, and highlighting considerations for future implementers.

Surprisingly, almost no known literature has examined how to implement OAAT therapy or its variations (i.e., single-session therapy) with children and youth, given the complexities of such services, including parental consent and procedures inherent within the integrated service delivery model. Young et al. (2012) noted select organizational considerations, including (1) updating organizational guidelines and policies, (2) having a dedicated role to oversee the implementation of OAAT therapy, and (3) ensuring staff have the support of managers. Young et al. (2012) also highlighted standard implementation best practices (i.e., ongoing consultations, senior leadership support, engaging champions) in the implementation of services for children and youth. Outside of these recommendations, researchers have not addressed pragmatic considerations (i.e., consent, integrated service models, changes in staffing roles) for implementing OAAT therapy in child and youth A&MHS, and particularly at a system level. This gap in literature presented challenges for the Department of Health in determining evidence-informed strategies and best practices for implementing OAAT therapy within child and youth A&MHS. As a result, the provincial working group elected to focus on providing OAAT therapy by rapid access appointments, allowing lower-barrier access to care. A scoping review on implementation science frameworks, models, and theories used in child, youth, and family services highlighted that the AIF-IS and CFIR are commonly used (Albers et al., 2017). Our study can serve as a starting point for applying best practices in implementation science (i.e., AIF-IS, CFIR, ERIC strategies) to implement OAAT therapy for children and youth.

The structure of implementation oversight and decision-making presented in Fig. 4 was vital in addressing contextual challenges during the implementation of OAAT therapy into child and youth A&MHS. Results from the risk and readiness assessments highlighted key concerns (e.g., insufficient staffing, lack of physical space) of frontline providers, OAAT clinical leads, and managers, which were discussed among the provincial working group. While staffing concerns were also raised as a potential barrier in the adult implementation (Harris-Lane et al., 2023), challenges accessing physical space to offer OAAT therapy sessions was unique to the child and youth sector. Allocating space for OAAT therapy sessions fell beyond the decision-making power of the provincial working group which precluded the use of the ERIC strategy “change physical structures” (Powell et al., 2015). A task force was established with senior government leaders with the ability to address spacing concerns. This innovative solution highlights the importance of engaged and motivated leadership.

In addition to the barriers identified in the readiness assessments, challenges emerged with the climate (Damschroder et al., 2022) associated with implementing OAAT therapy within an integrated service delivery model. Difficulties with interdisciplinary collaboration have been well-documented in previous literature (Vinicor, 1995), and are particularly common in integrated service delivery models (Nooteboom et al., 2021). As such, it was unsurprising that tensions surfaced between professions during the initial implementation. The provincial working group’s approach to educate provider groups on the respective roles in the operations and delivery of OAAT therapy aligns with interprofessional education—a best practice for collaboration (Atkins et al., 2017; Canadian Interprofessional Health Collaborative, 2010). This was further supported by scoping review on strategies to enhance interprofessional collaboration, as education on respective roles in the service delivery change was identified as a key strategy for improving communication and shared decision-making between professions (Sirimsi et al., 2022). Given the inherent alignment between integrated service delivery and SC2.0 frameworks, implementing OAAT therapy within a continuum of care requires enhanced interprofessional collaboration and a deeper understanding of each role within the system. Ultimately, strong interprofessional collaboration facilitates easier transitions in care across the continuum and ensures that children and youth receive the right service at the right time. Future implementers may consider other strategies detailed in the Sirimsi et al. (2022) review (e.g., develop a shared vision, set formal and informal meetings, and use structured guidelines).

A critical learning from this implementation was the value of completing a client journey mapping exercise to conceptualize ongoing changes to service delivery processes, particularly within complex systems. This technique has been used in past healthcare initiatives to increase clinical effectiveness, and improve patient experiences and healthcare delivery by creating a narrative timeline of what a patient would encounter as they navigate the system (Ly et al., 2021; McCarthy et al., 2020). The two most common reasons client journey mapping is used is for health service redesign and developing a more nuanced understanding of the client’s journey through the health system (Davies et al., 2023), both of which were present in the current study. In alignment with core components of SC2.0 (Carey et al., 2021), client journey mapping offers a client-centered approach to iterative improvements to service delivery processes. Client journey mapping also facilitates provider feedback and practice improvements by visualizing the client experience, and pinpointing areas of strength and weakness (Simonse et al., 2019). This exercise has benefitted multidisciplinary teams in conceptualizing challenges that may arise from differing perspectives and developing creative solutions to address shortcomings in pathways (McCarthy et al., 2020). McCarthy et al. (2020) demonstrated this in a case study where a client journey map exercise was used in collaboration between medical and technological teams when designing interventions. Collaboration between specialties allowed for a shared understanding of processes and the development of creative solutions to address limitations in patient pathways. Such interdisciplinary collaboration can be advantageous for integrated systems like New Brunswick, where healthcare and education providers work closely together to provide integrated services for children and youth.

An area for improvement highlighted within the client journey mapping exercise, and risk and readiness assessments, was the providers’ concern that youth may not call to book an OAAT therapy appointment. Previous research has highlighted the utility of providing online scheduling systems, as youth often prefer to schedule appointments online (DeJonckheere et al., 2020; Lawrence et al., 2024). The Government of New Brunswick took this into consideration during the implementation.

## Limitations and Future Research

Similar to Harris-Lane et al. (2023), results detailed in this manuscript should be interpreted with consideration of the following limitations. First, implementation processes in New Brunswick were retrospectively aligned with implementation science models, frameworks, and strategies. It is difficult to determine the extent of benefits that these frameworks could have in guiding similar implementations. Moreover, some elements of the implementation process overlapped or occurred in a different implementation stage than originally included in the AIF-IS (e.g., building an implementation team occurred in the installation rather than exploration stage). Notably, however, team members from Memorial University and Stepped Care Solutions brought implementation science expertise to the planning, preparation, and initial implementation, and implementation science in practice is not often linear and sequential (Nilsen, 2020). Future research on the implementation of OAAT therapy (and its equivalents) would benefit from a-priori application of implementation theories, frameworks, and models (i.e., using a logic model). The prospective use of the AIF-IS, CFIR, and ERIC strategies could help further mitigate potential risks, and prompt additional considerations on adapting strategies to best meet the needs of clients, providers, leadership, and the system. 

Second, fidelity to the OAAT therapeutic approach was not measured, which may impact the validity of findings. It is possible that OAAT therapy is not being delivered as intended at all sites. Future research should integrate measures of fidelity, given the importance (Gearing et al., 2011). Potential elements of an assessment of fidelity to an OAAT therapeutic approach could include the following: (1) was the session treated as a whole, complete in and of itself; (2) was the OAAT therapeutic approach introduced to new clients; (3) was a realistic outcome for the session established; (4) did the provider focus on and emphasize the client’s strengths; (5) was the client offered formal and informal resources and supports that span the continuum of care; (6) was an action plan developed; and (7) was the client reminded that they can access another session in the future if and when they need it.

Third, provider perceptions and client experiences were measured using self-report. While the response rate for the client satisfaction survey was low, the response rate among providers was high (> 50%) and likely representative. Our research highlights the overarching value of implementing OAAT therapy to improve access to addiction and mental healthcare for children and youth; however, it is imperative for future research to explore the implementation and delivery of OAAT therapy sessions through a lens of health equity and intersectionality. Specifically, through the lens of the health equity implementation framework (Woodward et al., 2021), future research should examine (1) if service users are representative of the New Brunswick population and if there are underrepresented groups, (2) barriers and facilitators to accessing OAAT therapy among diverse populations, and (3) if children and youth feel that they received appropriate, sensitive, and relevant care.

## Conclusions

This study begins to address a critical gap in the literature on the pragmatic process of implementing of OAAT therapy (and its equivalents) into child and youth A&MHS, particularly within an integrated approach to service delivery. Findings from the current study can be used to help inform future implementations and guide implementers in considering factors unique to child and youth A&MHS in alignment with evidence-based implementation science frameworks and models.

## Supplementary Information

Below is the link to the electronic supplementary material.

Supplementary file1 (DOCX 807 KB)

## Data Availability

De-identified data will be made available to researchers who provide a methodologically sound proposal for the purpose of achieving the aims of the approved proposal. Data sharing will be enacted with a data-transfer agreement between the sending and receiving institutions. Proposals should be directed to Joshua Rash (jarash@mun.ca).
