# Supplementary material for: Improving Access to Child and Youth Addiction and Mental Health Services in New Brunswick: Implementing One-at-a-Time Therapy Within an Integrated Service Delivery Model
Source: Int J Ment Health Addict. 2024 Jun 24;23(5):4096–117. doi: 10.1007/s11469-024-01339-4 (PMC12662875; doi:10.1007/s11469-024-01339-4)
Supplement: Supplementary file 1 — Supplementary file1 (DOCX 807 KB) [file 11469_2024_1339_MOESM1_ESM.docx]

**Supplemental Appendix A**

**
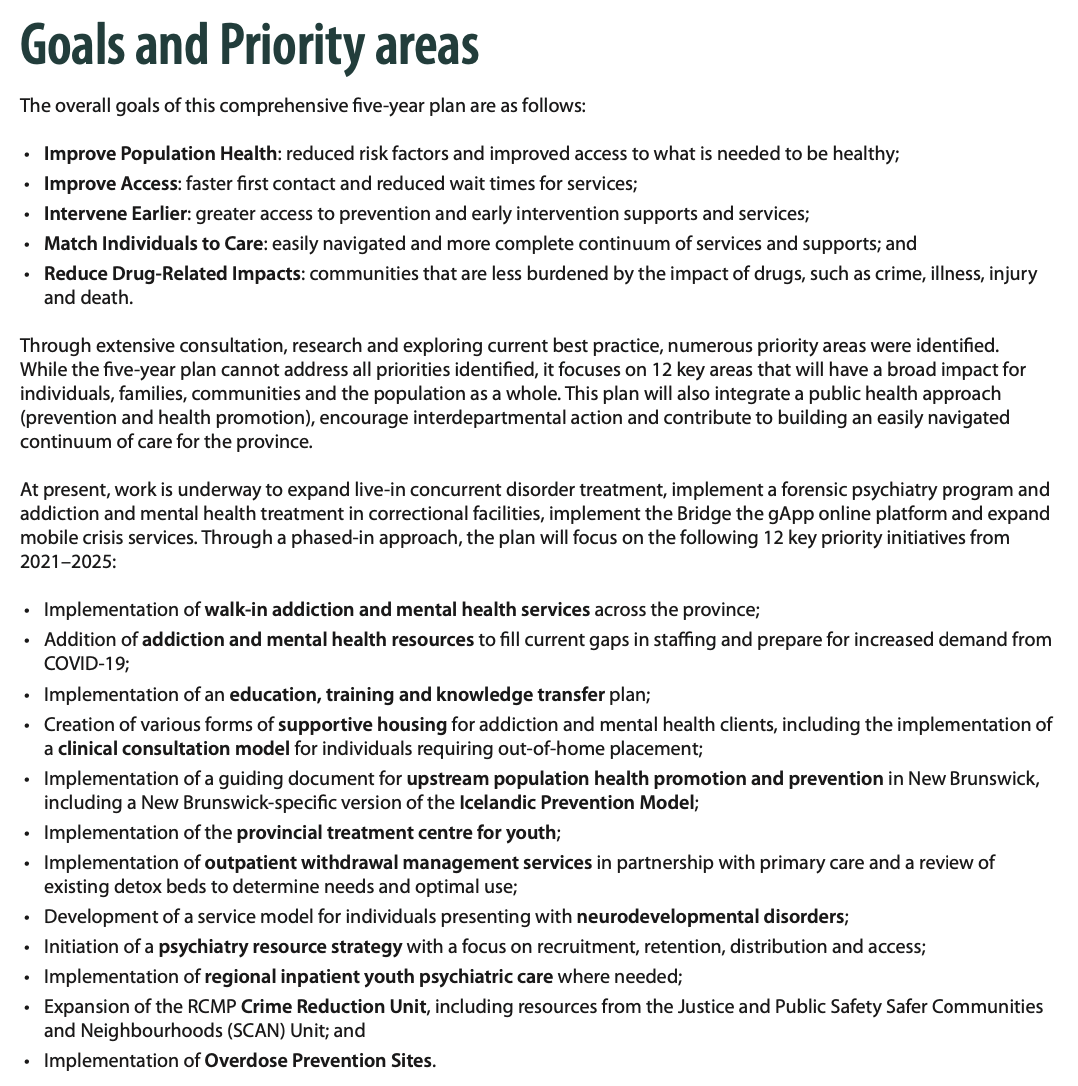
**

**Supplemental Appendix B**

**Introduction to Stepped Care 2.0 Course Outline**

The Stepped Care 2.0 course includes case studies, interactive content, journal entries, module summaries, and a post-course evaluation.

1. Module One
   1. Welcome to Stepped Care 2.0
   2. A New Approach to Mental Health Care
   3. What Is Stepped Care 2.0?
   4. Guiding Principles of Stepped Care 2.0
   5. The Stepped Care 2.0 Planning Framework (Nine-Steps)
   6. Three Factors of Stepped Care 2.0
   7. Navigating the Stepped Care 2.0 Continuum
2. Module Two
   1. The Core Components of Stepped Care 2.0
   2. Core Components 1–5: Model Design and Improvement
   3. Core Component 6: Clients Have Same Day Access to Multiple Levels of Care.
   4. Core Component 7: Treatment is Guided by Single-Session Thinking.
   5. Core Component 8: Treatment Planning is Flexible and Responsive by Strategically Reviewing Client Data and Making Data-Informed Adjustments as Needed.
   6. Core Component 9: Treatment Planning is Client-Centric.
   7. The Wellness Plan
   8. Making Decisions About Treatment Strategies
3. Module Three
   1. Recovery in Mental Health
   2. Moving Away from the Risk Paradigm
   3. Recovery Values
   4. Assessments in Stepped Care 2.0
   5. Strength-Based Practice
   6. Client-Centric Care

**Supplemental Appendix C**

**One-at-a-Time Therapy Course Outline**

The One-at-a-Time (OAAT) Therapy course includes case studies, interactive content, journal entries, module summaries, and a post-course evaluation.

1. Introduction to OAAT Therapy
   1. What Is OAAT Therapy?
   2. Adapting the OAAT Counselling Approach to Your Organization
   3. OAAT Therapy Approach in Stepped Care 2.0
   4. Research on OAAT Therapy
   5. Key OAAT Therapy Principles
   6. Developing a Single-Session Mindset
2. OAAT Therapy Session Structure
   1. Part 1: The Introduction
   2. Part 2: Establishing a Realistic Outcome
   3. Part 3: Identifying Strengths, Resources, and Previous Successes
   4. Part 4: Help the Client Achieve Their Realistic Outcome
   5. Part 5: The Closing
3. Resource Centre

**Supplemental Appendix D**

***RDS Subscales and Descriptions***

| **RDS Subscale** | **Subscale Description** |
| --- | --- |
| **Ability to Pilot** | The degree to which SC2.0 can be tested and improved in smaller settings before a full-scale implementation. |
| **Climate** | The overall feeling and tone within the organization. |
| **Compatibility** | The fit between SC2.0 and how the organization operates. |
| **Culture** | The alignment between SC2.0 and the organization’s norms and values. |
| **Innovativeness** | The organization’s openness and willingness to change. |
| **Inter-Organizational Relationships** | The organization’s relationships with other organizations and stakeholders implementing SC2.0. |
| **Intra-Organizational Relationships** | The relationships and level of coordination within the organization. |
| **Knowledge and Skills** | The skills and knowledge needed for staff to successfully use the SC2.0 model in their practice. |
| **Leadership** | The perceived effectiveness of the organization’s senior leaders. |
| **Observability** | The expectation to observe the intended impacts of SC2.0. |
| **Priority** | The importance of SC2.0 compared to the importance of other organizational operations. |
| **Program Champion** | The presence of individuals within the organization who are well connected and support the use of SC2.0 in their practice. |
| **Relative Advantage** | The advantage of SC2.0 compared to other innovative addiction and mental health models of care. |
| **Resource Utilization** | The organization’s ability to access and distribute financial resources to support SC2.0. |
| **Simplicity** | How simple or complex it will be to used SC2.0 in practice. |
| **Staff Capacity** | The workload of staff in relation to the capacity needed to carry out the SC2.0 model. |
| **Structure** | The structure and level of collaboration within the organization. |
| **Supportive Climate** | Presence of the necessary supports, processes, and resources to effectively enact SC2.0. |

**Supplemental Appendix E**

| 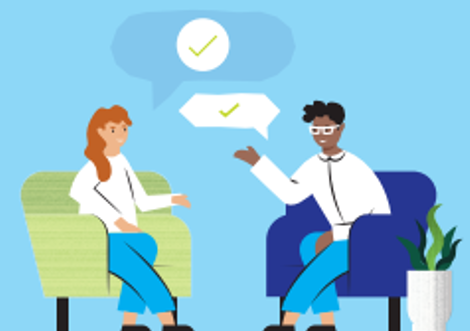 | **Child and Youth Team**  **One-at-a-time Therapy Client Satisfaction Survey**  **Small change leads to big change** |
| --- | --- |

| Date: |  | School Name: |  | Parent/Guardian present:  Yes ⬜ No ⬜ |
| --- | --- | --- | --- | --- |

1. **How upset were you about your problems before this counseling session?**

| V*ery upset*  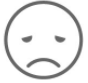 | *Upset*  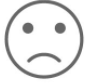 | *Not sure*  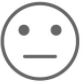 | *Somewhat upset*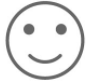 | *Not upset*  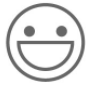 |
| --- | --- | --- | --- | --- |
| 1 | 2 | 3 | 4 | 5 |
|  |  |  |  |  |

1. **How upset are you about these problems after this counseling session?**

| V*ery upset*  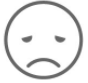 | *Upset*  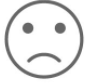 | *Not sure*  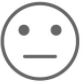 | *Somewhat upset*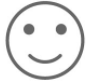 | *Not upset*  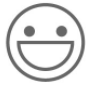 |
| --- | --- | --- | --- | --- |
| 1 | 2 | 3 | 4 | 5 |

1. **How confident do you feel in dealing with your problems?**

| *Not confident*  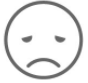 | *Somewhat Confident*  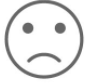 | *Not sure*  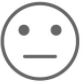 | *Confident*  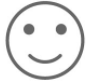 | *Very confident*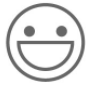 |
| --- | --- | --- | --- | --- |
| 1 | 2 | 3 | 4 | 5 |

1. **How satisfied are you with how your problems were addressed?**

| *Not satisfied*  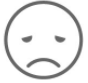 | *Somewhat satisfied*  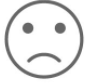 | *Not sure*  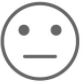 | *Satisfied*  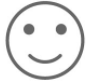 | *Very satisfied*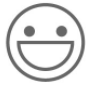 |
| --- | --- | --- | --- | --- |
| 1 | 2 | 3 | 4 | 5 |

1. **How satisfied are you with the plan you and the therapist developed?**

| Not satisfied  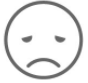 | *Somewhat satisfied*  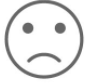 | *Not sure*  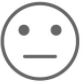 | *Satisfied*  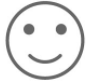 | *Very satisfied*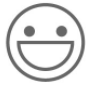 |
| --- | --- | --- | --- | --- |
| 1 | 2 | 3 | 4 | 5 |

**Other Comments: ______________________________________________________________**

**Supplemental Appendix F**

| **Exploration Stage** | | | | | |
| --- | --- | --- | --- | --- | --- |
| **Implementation Step** | **Description of New Brunswick Implementation Step** | **Timeframe** | **NIRN Components** | **CFIR Domain: Construct(s)** | **ERIC Strategies** |
| Review potential systems and models | The Department of Health (DoH) considered various known systems, models, and frameworks for addiction and mental health services and explored the similarities and differences, as well as advantages and disadvantages. This review led to the selection of Stepped Care 2.0 (SC2.0), as it has strong face validity and allows for a comprehensive and recovery-oriented continuum of services with varying levels of intensity. Further, SC2.0 focuses on rapid access to care and promotion of evidence-based practices. Its applicability in the development of a large-scale province-wide service delivery was also key in its selection. | 2018 – 2020 | -Identify and learn about other potential programs to address change needed.  -Assess fit and feasibility of options to address change needed. | Innovation Domain: 1) evidence-base; 2) relative advantage; 3) adaptability; 4) design |  |
| Partnerships with Memorial University and Stepped Care Solutions | The Government of New Brunswick formed partnerships with Memorial University and Stepped Care Solutions. These partnerships fostered implementation support and data collection on providers’ knowledge and beliefs about One-at-a-Time (OAAT) therapy and SC2.0, their self-efficacy, readiness, stage of change, work engagement and satisfaction, and competency in OAAT therapy and SC2.0. | Jan. 2021 |  | Individuals Domain: implementation facilitators | -Develop academic partnerships  -Seek guidance from experts in implementation |
| Release of Provincial 5-Year Action Plan | The inter-departmental 5-year action plan for mental health and addiction care was released and included priorities to create an integrated continuum of care and facilitate rapid access to services. | Feb. 2021 | -Scan or assess what is currently in place to address the change needed.  -Identify the option to implement. |  | -Conduct local consensus discussions  -Obtain formal commitments  -Mandate change  -Access new funding |
| **Installation Stage** | | | | | |
| **Implementation Step** | **Description of New Brunswick Implementation Step** | **Timeframe** | **NIRN Components** | **CFIR Domain: Construct(s)** | **ERIC Strategies** |
| Formation of core project team | The core project team was initially formed in June 2021, included membership from the directors responsible for child and youth services, a senior health consultant, change management specialist (refer to Harris-Lane et al., 2023), and project manager. This team helped lead the implementation of OAAT therapy across the province. Hiring a senior health consultant (Oct. 2021) was unique to the child and youth implementation, with the goal of: 1) serving as the point-contact for implementation leads; 2) monitoring readiness and risk; and 3) building sustainability. | June 2021;  Oct. 2021 | -Begin to build implementation team | Implementation Process Domain: teaming | -Facilitation  -Recruit for leadership  -Use an implementation advisor  -Develop and organize quality monitoring systems |
| Identify OAAT clinical leads | Clinical leads helped coordinate OAAT therapy services in their region. This included providing clinical support and guidance to providers who deliver the service, building an understanding of the service within the local community, playing a leadership role in the OAAT therapy Community of Practice, and promoting the role of OAAT therapy in a SC2.0 continuum of services.  The process of hiring OAAT leads began in July 2021, with all positions filled by the Spring of 2022 (installation and initial implementation stages) | July 2021 – Apr. 2022 | -Cultivate sponsors  -Secure and develop infrastructure resources and supports needed for the program/ practice. | Individuals Domain: 1) implementation leads; 2) implementation facilitators.  Inner Setting Domain: 1) relational connections; 2) communications; 3) culture. | -Identify and prepare champions  -Create new clinical teams  -Provide clinical supervision  -Distribute educational materials  -Facilitate relay of clinical data to providers  -Facilitation |
| Establish and educate Provincial Working Group (implementation team) | The provincial working group was comprised of leadership (i.e., directors, managers, and OAAT leads) and members of the core implementation team.  The provincial working group was responsible for: 1) reviewing and continuously adapting the formal project plan; 2) reviewing system processes and required changes to effectively implement OAAT therapy (i.e., intake processes and provider documentation); and 3) assessing organizational readiness and strategies use to mitigate barriers. | Oct. 2021 | -Built and revisit implementation team membership to ensure needed perspectives are present.  -Ensure implementation team has appropriate knowledge, skills, functions, and authority to support infrastructure development and improvement of the program/practice. | Individuals Domain: 1) high-level leaders; 2) mid-level leaders; 3) implementation leads; 4) implementation team members.  Implementation Process Domain: teaming | -Use advisory boards and workgroups  -Identify and prepare champions |
| Hold weekly Provincial Working Group meetings | The provincial working group met weekly throughout the implementation stages to fulfil their mandate with the formal project plan, system processes, and organizational readiness. | Oct. 2021 – Dec. 2022 | -Convene implementation team regularly to use data to critically examine and improve implementation  -Develop and use feedback loops between practitioners, leadership, community partners, and stakeholders to ensure effective communication. | Implementation Process Domain: 1) assessing needs; 2) assessing context; 3) planning; 4) tailoring strategies; 5) engaging; 6) reflecting & evaluating; 7) adapting | -Purposely re-examine the implementation  -Promote adaptability  -Facilitation  -Develop and organize quality monitoring systems |
| Adapt formal implementation plan | The core implementation team adapted the implementation plan developed for use in adult services to accommodate the unique context of child and youth services. The implementation plan included: 1) a project charter and change management strategy; 2) plan for training providers in OAAT therapy and SC2.0; and 3) communication plan. | Oct. 2021 | -Develop plan for implementation | Implementation Process Domain: 1) planning; 2) tailoring strategies; 3) adapting | -Develop a formal implementation blueprint  -Develop an implementation glossary  -Develop and implement tools for quality monitoring  -Develop educational materials |
| Beginning of ongoing risk and readiness assessments | Ongoing assessments of risk and readiness were completed through formal interviews with managers, check-ins with OAAT leads, and tracking questions and concerns about the implementation. | Oct. 2021 – Mar. 2022 | -Assess and create readiness for team, staff, and organization.  -Determine what is needed to optimize readiness and develop staff capacity, as well as organization and system changes needed. | Inner Setting Domain: 1) structural characteristics; 2) communications; 3) culture; 4) tension for change; 5) compatibility; 6) relative priority; 7) available resources  Implementation Process Domain: 1) assessing needs; 2) assessing context; 3) tailoring strategies | -Conduct local needs assessment  -Assess for readiness and identify barriers and facilitators.  -Tailor strategies  -Audit and provide feedback  -Promote adaptability |
| Providers complete online trainings | Providers completed online asynchronous courses in OAAT therapy and SC2.0. The online courses allowed staff to better understand the upcoming changes in the system and their role. | Nov. 2021 – Mar. 2021 | -Provide initial training for practitioners | Inner Setting Domain: access to knowledge and information | Conduct ongoing training |
| Revise operational guidelines and processes | Operational procedures and guidelines developed for adult service delivery were adapted to accommodate service delivery procedures in both the adult and child and youth service sectors. | Dec. 2021 – Aug 2022 | -Review and refine needed policies and procedures.  -Develop data systems (what data, how data will be collected, used, and shared). |  | -Purposely re-examine the implementation  -Promote adaptability  -Develop and implement tools for quality monitoring  -Change record systems |
| Host information sessions with providers | Information sessions for providers were delivered by the DoH Director of Mental Health and Addiction for Child and Youth services, change management specialist, and senior health consultant. These sessions facilitated learning about system changes associated with implementing OAAT therapy in a provincial SC2.0 framework, and provided an opportunity for providers to voice their questions and concerns. | Dec. 2021 – Jan. 2022 | -Provide initial training for providers. | Inner Setting Domain: access to knowledge and information | -Conduct educational meetings |
| Offer live training in OAAT therapy delivered by key field experts | Providers completed a live, two-day training, with a field expert in OAAT (Single-Session) therapy, to further their knowledge and abilities, and receive coaching | Jan. 2022 – Oct. 2022 | -Continue training/professional learning as needed. | Inner Setting Domain: access to knowledge and information  Implementation Process Domain: engaging | -Conduct ongoing training  -Make training dynamic  -Work with educational institutions |

| **Initial Implementation** | | | | | |
| --- | --- | --- | --- | --- | --- |
| **Implementation Step** | **Description of New Brunswick Implementation Step** | **Timeframe** | **NIRN Components** | **CFIR Domain: Construct(s)** | **ERIC Strategies** |
| Implement OAAT therapy into addiction and mental health services | Providers started delivering OAAT therapy to clients on waitlists and expanded to offering OAAT therapy to new referrals, as well as select clients on existing caseloads. | Apr. 2022 – Dec. 2022 | -Practitioners begin delivery/use of program/practice. | Implementation Process Domain: doing |  |
| Information sessions for community partners | Healthcare providers, education providers, and community partners experienced early tensions due to the need for an enhanced understanding of the referral process, and of the roles of themselves and their collaborators in the delivery of OAAT therapy. The core project team held education sessions with these stakeholders to clarify roles and expectations, and help mitigate these tensions. | Apr. 2022 – Oct. 2022 | -Continue training/professional learning as needed.  - Gather data and feedback through multiple sources including staff, practitioners, families, and stakeholders to monitor progress | Implementation Process Domain: 1) tailoring strategies; 2) engaging | -Conduct educational meetings |
| Task force formed | The core project team formed a task force with senior members of government leadership in healthcare and education to address challenges with insufficient spacing to offer OAAT therapy services to children and youth. | Apr. 2022 – Sept. 2022 | -Revisit implementation team membership to ensure needed perspectives.  -Secure and develop infrastructure resources and supports needed for the program/practice. | Implementation Process Domain: 1) assessing context; 2) planning; 3) tailoring strategies  Inner Setting Domain: available resources | -Involve executive boards  -Change physical structure and equipment |
| Assessing and tailoring data systems | The DoH started an initiative to transition from standardized manual reports of waitlist data to collecting and monitoring data through the Client Service Delivery System (CSDS). Implementing CSDS would allow for better capture of data on the impacts of implementation, and to monitor delivery of services (e.g., number of OAAT therapy sessions delivered). | May 2022 – Oct. 2022 | -Scan or assess what is currently in place to address the change needed  -Develop data systems (what data, how data will be collected, used, and shared). | Inner Setting Domain: structural characteristics (information technology infrastructure) | -Change record systems  -Develop and organize quality monitoring systems |
| Collect client feedback | Clients who received an OAAT therapy session were offered the opportunity to complete a client satisfaction survey at the end of the session. This feedback was reviewed for improvement of service delivery. | May 2022 – Dec. 2022 | -Gather data and feedback through multiple sources including staff, practitioners, families, and stakeholders to monitor progress | Implementation Process Domain: 1) engaging; 2) reflecting and evaluating | -Obtain and use feedback  from patients, consumers  and families  -Develop and implement tools for quality monitoring |
| Improve implementation and delivery of OAAT therapy | By monitoring system-level data and feedback from providers and clients, the provincial working group assessed the implementation and delivery of OAAT therapy and addressed challenges that arose. The feedback loop between the provincial working group (i.e., implementation team) and providers was vital in this process. | May 2022 – Dec. 2022 | -Use a process to develop improvement strategies through analysis of data and feedback.  -Refine implementation infrastructure (i.e., training, coaching, data systems, leadership supports and resources) based on data and feedback. | Implementation Process Domain: 1) reflecting and evaluating; 2) adapting | -Purposely re-examine the implementation |
| Client journey mapping | The core project team completed a client journey map exercise with regional managers, OAAT leads, and providers to conceptualize changes in service delivery processes, and identify potential barriers and facilitators to offering OAAT therapy sessions within existing policies | Jul. 2022 – Aug. 2022 |  | Implementation Process Domain: 1) assessing needs; 2) assessing context; 3) planning; 4) tailoring strategies | -Assess for readiness and identify barriers and facilitators  -Develop educational materials  -Model and simulate change |
| **Full Implementation** | | | | | |
| **Implementation Step** | **Description of New Brunswick Implementation Step** | **Timeframe** | **NIRN Components** | **CFIR Domain: Construct(s)** | **ERIC Strategies** |
| Offer a community of practice | OAAT leads began participating in a community of practice. Community of practice meetings foster continued professional development, allow for consultation with subject-matter experts, and provide a venue to highlight successes and address concerns. | Apr. 2022 – Present | -Continue the training and coaching supports to maintain skillful use of the program or practice.  -Monitor and improve implementation supports and resources as needed to sustain implementation. | Implementation Process Domain: 1) reflecting and evaluating; 2) adapting | -Capture and share local knowledge  -Organize clinician implementation team meetings  -Create a learning collaborative |
| Train newly hired staff | To ensure sustainability, a process was put in place to allow newly hired staff to receive training from the online asynchronous courses and complete the two-day live OAAT therapy trainings with a field expert. | Apr. 2022 – Present | -Continue the training and coaching supports to maintain skillful use of the program or practice. | Inner setting: access to knowledge and resources | -Conduct ongoing training |
| Supervisor Training | A supervisor training was held to help strengthen supervisory skills and develop greater consistency in supporting providers delivering OAAT therapy. | Sept. 2022 | -Monitor and improve implementation supports and resources as needed to sustain implementation. | Inner setting: access to knowledge and resources | -Recruit, designate, and train for leadership  -Use train-the-trainer strategies |
| Implement methods to measure fidelity for the delivery of OAAT therapy among providers | A fidelity measure of desired OAAT therapy practices was developed and adherence is presently being assessed. | Ongoing | -Continue to collect and use data for improvement (e.g., fidelity, program/process, outcome). | Implementation Process Domain: 1) reflecting and evaluating; 2) adapting | -Develop and implement tools for quality monitoring |
| Continue to review system data, operational guidelines, and stakeholder feedback | System data, operational guidelines, and stakeholder feedback will continue to be reviewed. | Ongoing | -Continue to collect and use data for improvement (e.g., fidelity, program/process, outcomes).  -Continue to use feedback loops with leadership, practitioners, staff, recipients and stakeholders to communicate about progress, improvement strategies and success. | Implementation Process Domain: 1) reflecting and evaluating; 2) adapting | -Obtain and use patients/consumers and family feedback |

**Supplemental Appendix G**

Example flow of service delivery map

**Before Implementing OAAT Therapy**

**
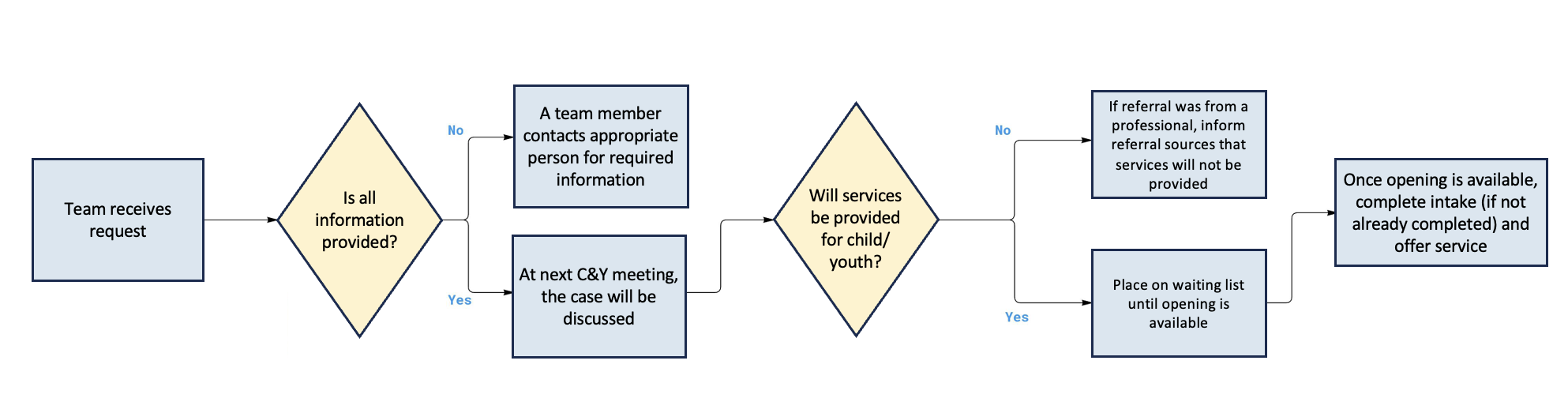
**

**After Implementing OAAT Therapy**

**
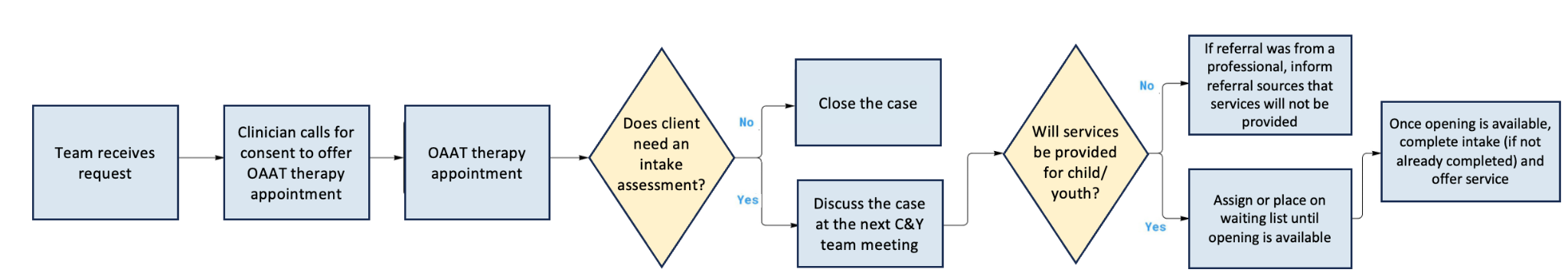
**

Client Journey Map

**
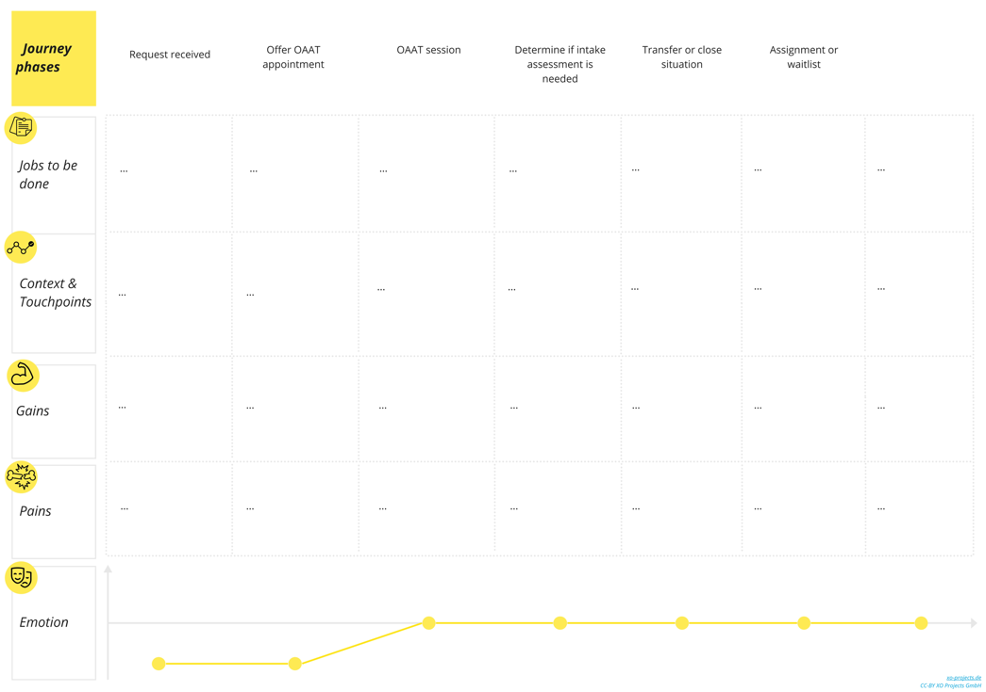
**
